# Comparison of calculated and experimental power in maximal lactate-steady state during cycling

**DOI:** 10.1186/1742-4682-11-25

**Published:** 2014-05-27

**Authors:** Thomas Hauser, Jennifer Adam, Henry Schulz

**Affiliations:** 1 Chemnitz University of Technology, Chemnitz, Germany; 2 Department of Internal Medicine/Cardiology, University of Leipzig, Heart Centre, Leipzig, Germany

**Keywords:** Maximal lactate-steady-state, Calculation, Lactate-production rate, Elimination of lactate

## Abstract

**Background:**

The purpose of this study was the comparison of the calculated (MLSS_C_) and experimental power (MLSS_E_) in maximal lactate steady-state (MLSS) during cycling.

**Methods:**

13 male subjects (24.2 ± 4.76 years, 72.9 ± 6.9 kg, 178.5 ± 5.9 cm, ${\dot{V}O}_{2\max}$: 60.4 ± 8.6 ml min^−1^ kg^−1^, ${\dot{V}\mathrm{La}}_{\text{max}}$: 0.9 ± 0.19 mmol l^-1^ s^-1^) performed a ramp-test for determining the ${\dot{V}O}_{2\max}$ and a 15 s sprint-test for measuring the maximal glycolytic rate (${\dot{V}\mathrm{La}}_{\text{max}}$). All tests were performed on a Lode-Cycle-Ergometer. ${\dot{V}O}_{2\max}$ and ${\dot{V}\mathrm{La}}_{\text{max}}$ were used to calculate MLSS_C_. For the determination of MLSS_E_ several 30 min constant load tests were performed. MLSS_E_ was defined as the highest workload that can be maintained without an increase of blood-lactate-concentration (BLC) of more than 0.05 mmol l^−1^ min^−1^ during the last 20 min. Power in following constant-load test was set higher or lower depending on BLC.

**Results:**

MLSS_E_ and MLSS_C_ were measured respectively at 217 ± 51 W and 229 ± 47 W, while mean difference was −12 ± 20 W. Orthogonal regression was calculated with r = 0.92 (p < 0.001).

**Conclusions:**

The difference of 12 W can be explained by the biological variability of ${\dot{V}O}_{2\max}$ and ${\dot{V}\mathrm{La}}_{\text{max}}$. The knowledge of both parameters, as well as their individual influence on MLSS, could be important for establishing training recommendations, which could lead to either an improvement in ${\dot{V}O}_{2\max}$ or ${\dot{V}\mathrm{La}}_{\text{max}}$ by performing high intensity or low intensity exercise training, respectively. Furthermore the validity of ${\dot{V}\mathrm{La}}_{\text{max}}$ -test should be focused in further studies.

## Introduction

Over the last 35 years, incremental graded exercise tests have been established for detecting endurance performance on the basis of a lactate-performance curve and the application of several different lactate-threshold concepts [1]. Most of these lactate concepts have the aim to approximate the power output achieved at maximal lactate-steady-state (PMLSS), which is one criterion of endurance performance [1,2]. PMLSS is defined as the highest workload where lactate-formation and lactate-elimination in the muscle cell are maintained at a steady-state [2-4]. However, Hauser et al. [5] compared the power at "onset of blood lactate accumulation" (OBLA) [6,7], the "individual anaerobic threshold" (IAT) [8] and the " + 1.5 mmol·l^−1^ lactate model" [9] with power in MLSS, measured during 30-minutes constant load tests. They found high significant correlations between OBLA and MLSS: r = 0.89 (mean difference −7.4 W); IAT and MLSS: r = 0.83 (mean difference 12.4 W), +1.5 mmol·l^−1^ lactate model and MLSS: r = 0.88 (mean difference −37.4 W). However, based on Bland-and-Altman, the comparison of power of all threshold-concepts with power in MLSS showed large individual differences, which deceive the high regression coefficients and small mean differences between these methods.

Furthermore, it is problematical that lactate-threshold concepts are based solely on the blood-lactate-concentration (BLC), which is mainly influenced by lactate formation, −transport, −diffusion and -elimination. Therefore, BLC may not represent the true metabolic processes occurring within the muscle cell. Mader [10,11] and Bleicher et al. [12] have previously suggested that the same lactate-performance-curve may result from different combinations of maximal oxygen uptake (${\dot{V}O}_{2\max}$) and maximal lactate production rate (${\dot{V}\mathrm{La}}_{\text{max}}$). Furthermore, the shift of a lactate-performance curve could also be achieved by changing ${\dot{V}O}_{2\max}$ or ${\dot{V}\mathrm{La}}_{\text{max}}$ separately.

Indeed, Bleicher et al. [12] verified, that two different athletes, (soccer and track), had exactly the same velocity for onset of blood lactate accumulation (OBLA) of 4.4 m s^−1^, yet the individual parameters of ${\dot{V}O}_{2\max}$ and ${\dot{V}\mathrm{La}}_{\text{max}}$ were higher for the soccer player when compared to the track athlete (${\dot{V}O}_{2\max}$: 70 vs. 63 ml min^−1^ kg^−1^; ${\dot{V}\mathrm{La}}_{\text{max}}$ 0.93 vs. 0.65 mmol l^−1^ s^−1^, respectively). That confirms, therefore that identical MLSS could be originate by completely different combinations of ${\dot{V}O}_{2\max}$ - and ${\dot{V}\mathrm{La}}_{\text{max}}$ -values. Using either ${\dot{V}O}_{2\max}$, ${\dot{V}\mathrm{La}}_{\text{max}}$ or BLC alone, it is not possible to explain differences of PMLSS between two athletes or the effects of training on the MLSS. As such, it would be beneficial to understand, how MLSS is controlled by glycolysis and oxidative phosphorylation within the muscle cell.

**Figure 1:**
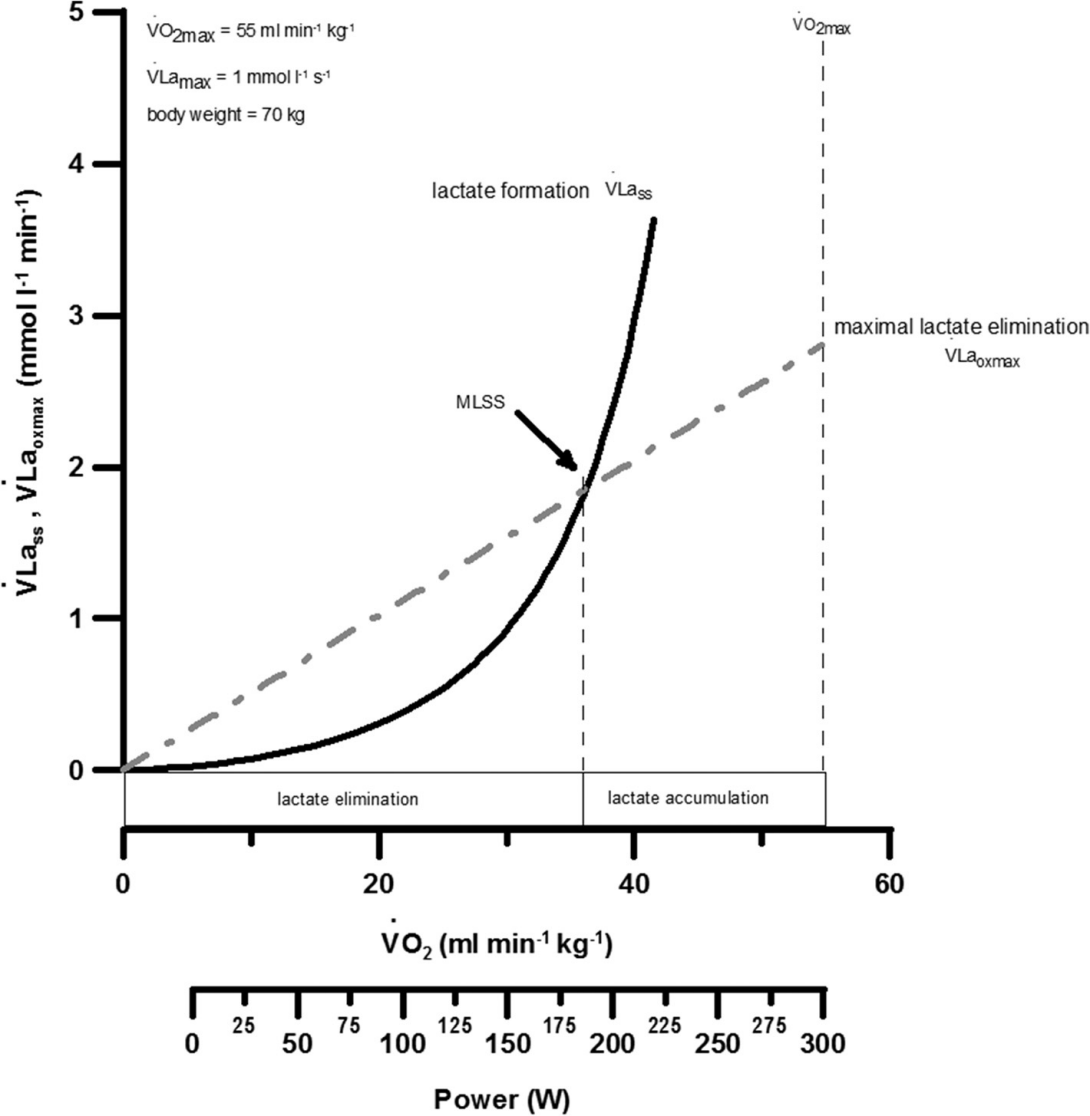
**Presented are the parameters of “gross” lactate formation (**${\dot{V}\mathrm{La}}_{\mathrm{ss}}$**).** The maximal elimination of lactate (${\dot{V}\mathrm{La}}_{\mathrm{oxmax}}$) and maximal-lactate-steady-state (PMLSS) independence of ${\dot{V}O}_{2}$ steady-state (${\dot{V}O}_{2\mathrm{ss}}$). All parameters are dictated by the ${\dot{V}O}_{2}$ steady-state (Figure modified from Mader and Heck [3]).

To explain the metabolic background of MLSS, Mader and Heck [3] introduced “A theory of the metabolic origin of anaerobic threshold”. The authors published a mathematical description of the metabolic response, based on measured values, exemplarily for a single muscle cell. They focussed on the activation of glycolysis (as the lactate production system) and on the oxidative phosphorylation (as the combustion system for lactate). Mader and Heck [3] argued that on the basis of Michaelis-Menten kinetics, it would be possible to calculate at the same time both, the rate of lactate-formation by glycolysis and its rate of lactate elimination by the oxidative phosphorylation, depending on a constant workload. These authors subsequently defined PMLSS as the crossing point at which the lactate-formation (${\dot{V}\mathrm{La}}_{\mathrm{ss}}$) exactly equates to the maximal-elimination-rate of lactate (${\dot{V}\mathrm{La}}_{\mathrm{oxmax}}$) as shown in Figure 1.

The present study, therefore, hypothesised firstly that it would be possible to calculate the PMLSS using the method by Mader and Heck [3] and secondly that knowledge of ${\dot{V}O}_{2\max}$ and ${\dot{V}\mathrm{La}}_{\text{max}}$ (and their interaction) would help to better understand the mechanisms of the MLSS. These outcomes could provide a benefit compared to lactate-threshold concepts and to time-extensive 30 min constant-load tests.

## Methods

### Study sample

13 male subjects (age: 24.2 ± 4.76 yr, weight: 72.9 ± 6.9 kg, height: 178.5 ± 5.9 cm, ${\dot{V}O}_{2\max}$: 60.4 ± 8.6 ml·min^−1^·kg^−1^, ${\dot{V}\mathrm{La}}_{\text{max}}$: 0.9 ± 0.19 mmol l^-1^ s^-1^) with different endurance levels participated in this study (training volume: n = 4 between 10 and 14 hours/week, n = 7 from 2 to 8 hours/week, n = 2 no sport). All subjects were informed about the aims of the study and subsequently provided written consent in accordance with the declaration of Helsinki [13].

### Procedure

All tests were performed on a Lode Excalibur Sport Ergometer (Lode, Groningen, NL). At the beginning of this investigation subjects performed, in a random order, a ${\dot{V}\mathrm{La}}_{\text{max}}$ test for detecting the maximal glycolytic rate and a ${\dot{V}O}_{2\max}$ test for detecting the maximal aerobic performance. Using the method introduced by Mader and Heck [3], PMLSS_C_ was calculated on the basis of the individual ${\dot{V}O}_{2\max}$, ${\dot{V}\mathrm{La}}_{\text{max}}$ and body weight. PMLSS_C_ was used for the first constant-load-test and several 30 min constant load-tests were undertaken to detect PMLSS_E_. Each test was performed on different days.

### $\dot{V}{\mathrm{La}}_{\text{max}}-\mathrm{test}$

In order to detect ${\dot{V}\mathrm{La}}_{\text{max}}$ the subjects performed a sprint-test lasting 15 s which consisted of a 12 min warm-up period with a constant load set at 1.5 times of the individual body weight, followed by a second exercise bout with a constant load of 50 W for ten minutes. Directly after finishing the warm up phase, two blood samples were obtained from the earlobe in order to measure the lactate-concentration before the test. Following a countdown of 3 s the subjects began pedalling maximally in the seated position, with pedalling frequency being maintained at 130 rpm. The subjects had to retain the power output as long as possible. Blood samples were then immediately drawn and at every 60 s until the 9^th^ min after the end of the test, to determine the maximum-post-exercise-lactate. ${\dot{V}\mathrm{La}}_{\text{max}}$ was calculated according to Equation 1 [14]:

$$\dot{V}{\mathrm{La}}_{\text{max}}=\frac{{\mathrm{La}}_{\mathrm{maxPost}}-{\mathrm{La}}_{\mathrm{Pre}}}{t_{\mathrm{test}}-t_{\mathrm{alac}}}$$

**Equation 1:** Calculation of maximal glycolytic rate.

Abbreviations are as follows: La_maxPost_ = Maximal Post Exercise Bloodlactate, La_Pre_ = Bloodlactate before test, t_test_ = test duration = 15 sec, t_alac_ = alactic time interval

The alactic time interval (t_alac_) was defined as the time from the beginning of the sprint (0 sec) to when the maximum power decreases by 3.5%.

### $\dot{V}O_{2\max}-\mathrm{Test}$

Subjects performed a ramp-test for measuring ${\dot{V}O}_{2\max}$ breath-by-breath (Oxycon Pro, Jäger, Höchberg, Germany) which included a warm up of 10 minutes at a constant load corresponding to 1.5 times of the participant’s body-weight, followed by a period of 2 min at a constant load of 50 W. The workload at the beginning of the test was set to 50 W for 2 min and was increased by 25 W every 30 s. The test was finished when subjects reached physically exhaustion, complaints of shortness of breath, dizziness or other physical complaints that unabled them proceeding the test [15]. ${\dot{V}O}_{2\max}$ was calculated by the mean of all ${\dot{V}O}_{2}$ -values measured within the last 30s of the test.

### Calculation of PMLSS_C_

#### Step 1: Biochemical elementary background

In order to identify PMLSS_C_, the activity of glycolysis (${\dot{V}\mathrm{La}}_{\mathrm{ss}}$) and oxidative phosphorylation (${\dot{V}O}_{2\mathrm{ss}}$) must be known [3,11]. Activation of ${\dot{V}\mathrm{La}}_{\mathrm{ss}}$ and ${\dot{V}O}_{2\mathrm{ss}}$ can be separately expressed by using the Michaelis-Menten kinetics (Equation 2) that is generally characterised by the activation of a single enzyme depending on a substrate and the maximal performance of glycolysis and oxidative phosphorylation, which is represented by ${\dot{V}\mathrm{La}}_{\text{max}}$ and ${\dot{V}O}_{2\max}$ respectively. The K_M_ which represents 50% of maximal activity rate must also be known.

$${\dot{V}}_{0}=\frac{{\dot{V}}_{\text{max}}}{{1+K_{M}/\left[S\right]}^{n}}$$

**Equation 2:** Elementary equation of Michaels-Menten-kinetics, where activation of an enzyme-substrate-complex (${\dot{V}}_{0}$) depends on maximal performance (${\dot{V}}_{\text{max}}$), 50%-activity-constant (K_M_) and substrate (S).

It is mostly agreed that under nomoxic conditions the main regulating substrate (S) for the activation of ${\dot{V}O}_{2\mathrm{ss}}$ and ${\dot{V}\mathrm{La}}_{\mathrm{ss}}$ is the level of free ADP concentration [3,11,16,17]. With an increase of the workload and therefore a higher demand of ATP, ADP-concentration rises exponentially within the muscle towards ${\dot{V}O}_{2\mathrm{ss}}$ and ${\dot{V}\mathrm{La}}_{\mathrm{ss}}$[11].

#### Step 2: Activation of oxidative phosphorylation (${\dot{V}O}_{2\mathrm{ss}}$)

According to Mader [11] and Heck [3]${\dot{V}O}_{2\mathrm{ss}}$ can be assessed by using Hill equation (Equation 2) as a function of free ADP and ${\dot{V}O}_{2\max}$. The 50%-activity-rate-constant of ${\dot{V}O}_{2\mathrm{ss}}$ (Ks1) is related to the exponent of ADP, which must be greater than 1.0 [3,11,18] otherwise it is not possible to calculate an appropriate activation of ${\dot{V}O}_{2}$[3,11]. The exponent may reside in the range of 1.4 to 2 [17]. In the present paper an exponent of 2 was used, which leads to a 50% activity constant related to free ADP-concentration of 0.2512 mmol/kg of (0.2512)^2^ mmol/kg [3]. Therefore Ks1 was set to (ADP)^2^ = (0.2512)^2^ = 0.0631 [3,19].

$$\dot{V}O_{2\mathrm{ss}}=\frac{{\dot{V}O}_{2_{\text{max}}}}{{1+\mathrm{Ks}1/\left[\mathrm{ADP}\right]}^{2}}$$

**Equation 3:** Transformed equation of Michaels-Menten-kinetics to calculate the activation of oxidative phosphorylation (${\dot{V}O}_{2\mathrm{ss}}$) – depending on maximal oxygen uptake (${\dot{V}O}_{2\max}$), 50%-activity-constant (Ks1) and substrate (ADP).

#### Step 3: Activation of glycolysis (${\dot{V}\mathrm{La}}_{\mathrm{ss}}$)

${\dot{V}\mathrm{La}}_{\mathrm{ss}}$ mainly depends on the activation of the enzyme phosphofructokinase (PFK), which is activated by free ADP and AMP [3,11,18,20]. AMP amplifies the activity of glycolysis in addition to ADP which leads to an exponent of 3 [3,11]. Equation 4 describes the activation of ${\dot{V}\mathrm{La}}_{\mathrm{ss}}$ as a function of free ADP and ${\dot{V}\mathrm{La}}_{\text{max}}$. The 50%-activity-rate-constant of ${\dot{V}\mathrm{La}}_{\mathrm{ss}}$ (Ks2) due to PFK at ADP^3^ of 1.1 mmol/kg leads to Ks2 of 1.331 [3].

$$\dot{V}{\mathrm{La}}_{\mathrm{ss}}=\frac{{\dot{V}\mathrm{La}}_{\text{max}}}{1+\mathrm{Ks}2/{\left[\mathrm{ADP}\right]}^{3}}$$

**Figure 2:**
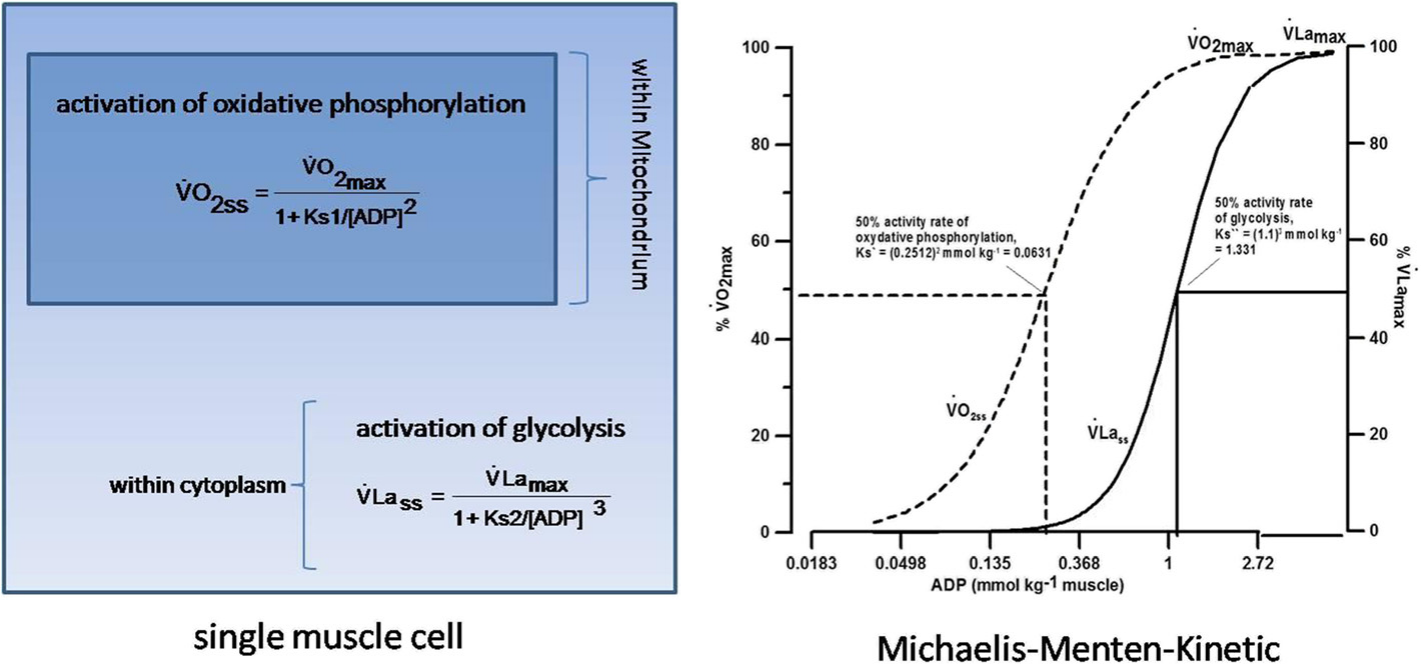
**Steady-state activation and 50% activity rate of oxidative phosphorylation (**$\dot{V}O_{2\mathrm{ss}}$**, Ks1 = 0.0631) and glycolysis (**$\dot{V}La_{\mathrm{ss}}$**, Ks2 = 1.331).** Data expressed as percentage of ${\dot{V}O}_{2\max}$ and ${\dot{V}\mathrm{La}}_{\text{max}}$ respectively, with respect to free ADP concentration (mmol/kg_m_). Modification from Mader and Heck [3].

**Equation 4:** Transformed equation of Michaels-Menten-kinetic to calculate the activation of glycolysis (${\dot{V}\mathrm{La}}_{\mathrm{ss}}$) - depending on maximal glycolytic rate (${\dot{V}\mathrm{La}}_{\text{max}}$), 50%-activity-constant (Ks2) and substrate (ADP) (Figure 2).

#### Step 4: Calculation of Lactate-elimination-rate depending on ${\dot{V}O}_{2\mathrm{ss}}$

The oxidation of lactate primary occurs within the active muscle. ${\dot{V}\mathrm{La}}_{\mathrm{oxmax}}$ is a linear function (Equation 5) of the current ${\dot{V}O}_{2}$[3,21]. Furthermore it not only depends on the amount of oxidized pyruvate/lactate per unit O_2_, which lies at 0.02049 mmol lactate/ml O_2_ but also on the distribution volume that was set to 0.4 in the present paper [3].

$$\dot{V}{\mathrm{La}}_{\mathrm{oxmax}}=\frac{\mathrm{lactate}‒\mathrm{equivalent}}{\mathrm{lactate}\;\mathrm{distribution}\;\mathrm{volume}}*\dot{V}O_{2\mathrm{ss}}=\frac{0.02049}{0.4}*\dot{V}O_{2\mathrm{ss}}$$

**Equation 5:** Calculation of maximal lactate elimination rate (${\dot{V}\mathrm{La}}_{\mathrm{oxmax}}$) – depending on lactate equivalent, lactate distribution volume and activity of oxidative phosphorylation.

However, there is no simple procedure to measure ADP-concentration and thus the activity rates of ${\dot{V}\mathrm{La}}_{\mathrm{ss}}$ and ${\dot{V}O}_{2\mathrm{ss}}$ in a daily endurance performance analysis. For an application of the model as a tool of endurance performance testing, ${\dot{V}\mathrm{La}}_{\mathrm{ss}}$ and ${\dot{V}O}_{2\mathrm{ss}}$ must be calculated without measuring the free ADP-concentration. This is possible when the mentioned equations are transposed from ADP in ${\dot{V}O}_{2\mathrm{ss}}$ depended equations.

#### Step 5: Transformation from ADP depended equations into ${\dot{V}O}_{2\mathrm{ss}}$ depended equations

During training or testing, ${\dot{V}O}_{2\mathrm{ss}}$ can easily be measured by spirometry-devices or determined by a calculation (Equation 6), which is based on a linear function between ${\dot{V}O}_{2\mathrm{ss}}$ and the workload [3].

$$\dot{V}O_{2\mathrm{ss}}=\frac{\left(P*\mathrm{Ks}4\right)+\left(\mathrm{bodyweight}*\dot{V}O_{2\mathrm{rest}}\right)}{\mathrm{bodyweight}}$$

**Equation 6:** Calculation for the activity of oxidative phosphorylation (${\dot{V}O}_{2\mathrm{ss}}$) as dictated by workload (P) and bodyweight.

If ${\dot{V}O}_{2\mathrm{ss}}$ is known or easily fit from 1 to ${\dot{V}O}_{2\max}$, Equation 2 can be rearranged in Equation 7. Therefore ADP-concentration can be calculated for a special workload depending on ${\dot{V}O}_{2\mathrm{ss}}$ and ${\dot{V}O}_{2\max}$, in the form of:

$$\left[\mathrm{ADP}\right]=\sqrt[{2}]{\frac{\mathrm{Ks}2*\dot{V}O_{2\mathrm{ss}}}{\left(\dot{V}O_{2\max}-\dot{V}O_{2\mathrm{ss}}\right)}}$$

**Equation 7:** Calculation of free ADP-concentration with respect to activated oxidative phosphorylation (${\dot{V}O}_{2\mathrm{ss}}$) and maximal oxygen uptake (${\dot{V}O}_{2\max}$).

After replacing the term ADP in Equation 3 with the right term of Equation 7, ${\dot{V}\mathrm{La}}_{\mathrm{ss}}$ can be calculated as a function of ${\dot{V}O}_{2\mathrm{ss}}$ using Equation 8.

$$\dot{V}La_{\mathrm{ss}}=\frac{60*\dot{V}La_{\text{max}}}{1+{\left(\frac{\mathrm{Ks}2}{\sqrt{\frac{\mathrm{Ks}1*\dot{V}O_{2\mathrm{ss}}}{\dot{V}O_{2\max}-\dot{V}O_{2\mathrm{ss}}}}}\right)}^{3}}$$

**Equation 8:** Calculation of glycolysis activity with respect to activated oxidative phosphorylation (VO_2ss_) and maximal glycolytic rate (${\dot{V}\mathrm{La}}_{\text{max}}$).

Furthermore, ${\dot{V}\mathrm{La}}_{\mathrm{oxmax}}$ can also be calculated as demonstrated in Equation 5.

#### Step 6: Calculation of PMLSS_C_ depending on ${\dot{V}O}_{2\mathrm{ss}}$

The empirical determined values of ${\dot{V}\mathrm{La}}_{2\max}$, ${\dot{V}\mathrm{La}}_{\text{max}}$ and body weight are needed in order to calculate PMLSS_C_. MLSS is defined at the power at which lactate formation exactly equates to the maximal lactate elimination rate. Mathematically, this means $\dot{V}{\mathrm{La}}_{\mathrm{ss}}=\dot{V}{\mathrm{La}}_{\mathrm{oxmax}}$. By using Equation 9, ${\dot{V}O}_{2\mathrm{ss}}$ in PMLSS can be calculated as:

$$0=\dot{V}{\mathrm{La}}_{\mathrm{ss}}-\dot{V}{\mathrm{Laox}}_{\text{max}}=\frac{60*\dot{V}{\mathrm{La}}_{\text{max}}}{1+{\left(\frac{\mathrm{Ks}2}{\sqrt{\frac{\mathrm{Ks}1*\dot{V}O_{2\mathrm{ss}}}{\dot{V}O_{2\max}-\dot{V}O_{2\mathrm{ss}}}}}\right)}^{3}}-\frac{0.02049}{{\mathrm{Vol}}_{\mathrm{rel}}}*\dot{V}O_{2\mathrm{ss}}$$

**Equation 9:** Calculation in the activity of glycolysis with respect to the activation oxidative phosphorylation (${\dot{V}O}_{2\mathrm{ss}}$) and maximal glycolytic rate (${\dot{V}\mathrm{La}}_{\text{max}}$).

Only Equation 9 has to be used to calculate MLSS. However, there is no analytic solution for the calculation of ${\dot{V}O}_{2\mathrm{ss}}$ in Equation 9. Therefore, a numerical approximation such as the numerical interval bisection method or multiple mathematical optimized methods, has to be used, as implemented in computer software. If ${\dot{V}O}_{2\mathrm{ss}}$ in PMLSS_C_ could be determined, PMLSS_C_ can be calculated by using Equation 10.

$${\mathrm{PMLSS}}_{C}=\frac{\left(\dot{V}O_{2\mathrm{ss}}*\mathrm{bodyweight}\right)-\left(\mathrm{bodyweight}*\dot{V}O_{2\mathrm{rest}}\right)}{\mathrm{Ks}4}$$

**Equation 10:** Calculation of power in MLSS (PMLSS_C_) depending on the activity of oxidative phosphorylation (${\dot{V}O}_{2\mathrm{ss}}$), bodyweight and oxygen/workload-constant (Ks4).

Therefore the relation between ${\dot{V}O}_{2}$ and power expressed as Ks4 must be known. In the present paper Ks4 was set to a constant value of 11.7 O_2_/W [3].

### Constant load tests

Subjects performed at least two 30 min constant load exercise tests at a cadence of 70–80 rpm for determination the PMLSS_E_[2]. The first constant-load test according to PMLSS_C_ started after a warm-up of 3 minutes at a power corresponding to 60% of the PMLSS_C_ rate. Blood samples were taken during rest, after 4 and 8 min, and at subsequent 2 min intervals until the end of the test. The PMLSS_E_ was defined as the highest workload that can be maintained without an increase of blood-lactate-concentration of more than 0.05 mmol·l^−1^·min^−1^ during the last 20 minutes of the test. Depending on blood-lactate-concentration, power in the next constant load test was set higher or lower by 10 W.

### Statistical analysis

All data were analyzed using the software SPSS version 14. Descriptive statistics were calculated from the data (means, standard deviations (SD), minimum and maximum values). Normal distribution was verified using the Shapiro-Wilk-Test. Relationship between variables was investigated using orthogonal regression and correlation. The level of significance was set at α = 0.05 for all analyses.

## Results

**Figure 3:**
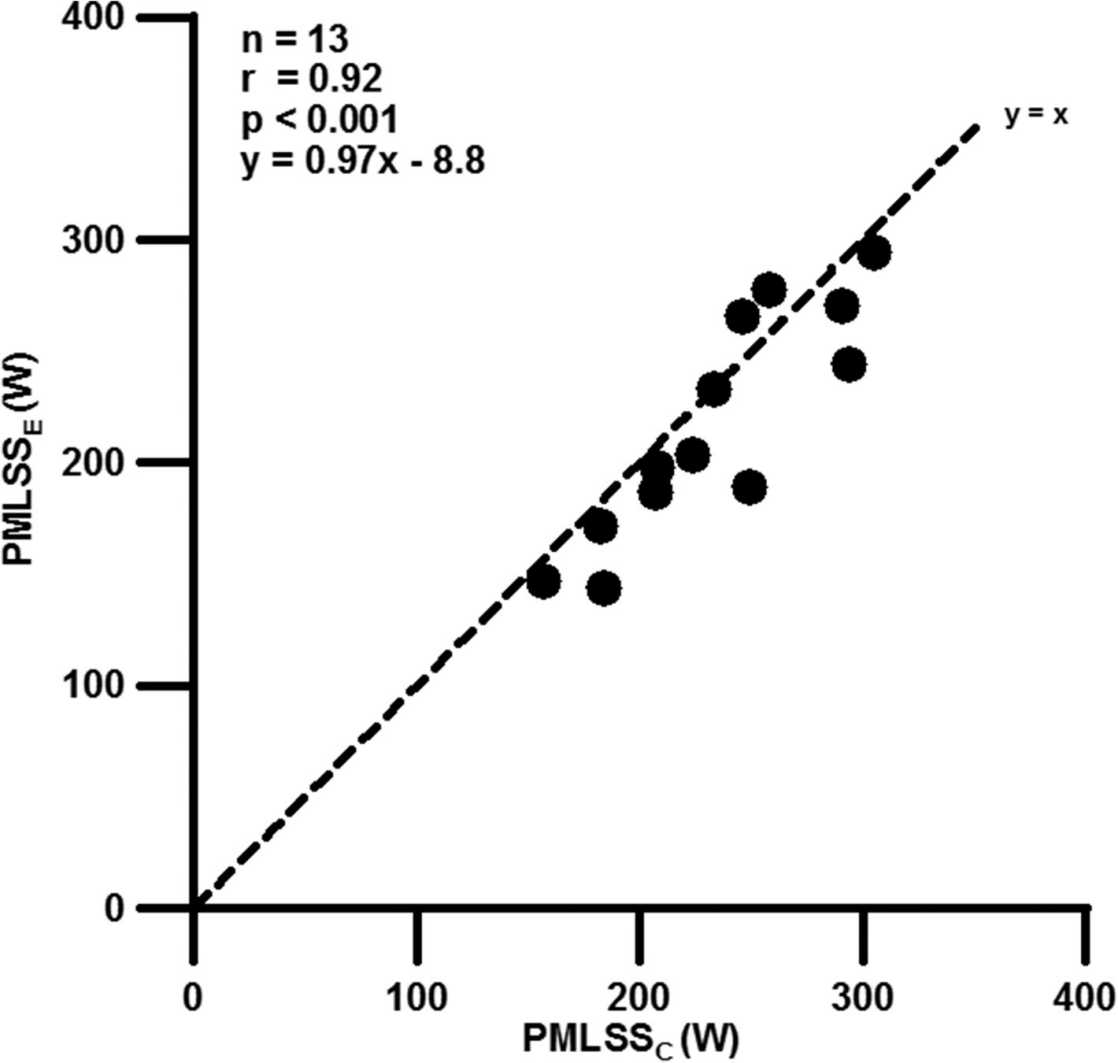
**Correlation and orthogonal regression of PMLSS**_**E**_** and PMLSS**_**C**_** (r = 0.92; p** ≤ **0.001).**

Descriptive values of ${\dot{V}\mathrm{La}}_{\text{max}}$, ${\dot{V}O}_{2\max}$, bodyweight, PMLSS_C_ and PMLSS_E_ are presented in Table 1. Furthermore, high significant correlation between PMLSS_E_ and PMLSS_C_ (r = 0.92; p < 0.001) (Figure 3) and PMLSS_E_ and ${\dot{V}O}_{2\max}$ (r = 0.84; p < 0.001) were found. ${\dot{V}\mathrm{La}}_{\text{max}}$ shows no correlation with PMLSS_E_ (r = −0.2; p > 0.05). The mean difference between PMLSS_C_ and PMLSS_E_ was 12 W ± 20 W.

**Table 1:** Results of maximum metabolic performance tests and calculated and experimental power in maximal lactate-steady state

**Subject**	${\dot{V}\mathrm{La}}_{\text{max}}$** (mmol·l**^**−1**^**·s**^**−1**^**)**	${\dot{V}O}_{2\text{max}}$** (ml·min**^**−1**^**·kg**^**−1**^**)**	**Bodyweight (kg)**	**PMLSS**_**C**_**(W)**	**PMLSS**_**E**_** (W)**	**Difference PMLSS**_**C**_**- PMLSS**_**E**_** (W)**
1	0.87	70.4	58.8	233	233	0
2	0.67	69.7	70.65	294	244	50
3	0.78	68.4	78.65	305	295	10
4	0.89	64.8	70.00	246	266	−20
5	1.39	61.0	66.2	182	172	10
6	0.74	60.0	62.7	208	198	10
7	1.02	55.8	76.65	207	187	20
8	0.98	48.0	71.9	157	147	10
9	0.98	56.6	80.1	224	204	20
10	1.07	71.3	75.8	291	271	20
11	0.74	47.1	78.55	184	144	40
12	0.94	62.7	78.35	258	278	−20
13	0.81	49.0	79.00	190	180	10
$\bar{x}$ ± s	0.91 ± 0.18	60.4 ± 8.6	72.9 ± 6.8	229 ± 47	217 ± 51	12 ± 20
min	1.39	71.3	80.1	305	295	50
max	0.67	47.1	58.8	157	144	−20

Abbreviations are as follows: min - minimum, max - maximum, PMLSS_C_ - power in calculated maximal lactate-steady-state, PMLSS_E_ - power in experimental maximal lactate-steady-state, SD – standard deviation, ${\dot{V}\mathrm{La}}_{\text{max}}$ - maximal lactat production rate, ${\dot{V}O}_{2\max}$ - maximum oxygen consumption at maximum load.

## Discussion

The aim of the present investigation was to compare the calculated and experimentally determined power output in MLSS. The comparison of PMLSS_C_ and PMLSS_E_ showed a highly significant correlation (0.92), with only a mean difference of 12 W ± 20 W between the two methods. The results of the present paper accords to previous comparisons between the different lactate-concepts and MLSS. It is well known that different lactate threshold concepts approximate in average MLSS rather well. Van Schuylenbergh et al. [22] published highly significant correlations between MLSS and OBLA and the Dmax method (r = 0.94 and r = 0.89, respectively). Heck [4] also evaluated correlations between MLSS and OBLA and individual anaerobic threshold of r = 0.92 and r = 0.87, respectively. However, as already mentioned, the investigation of Hauser et al. [5] showed large individual differences comparing power of threshold-concepts with power in MLSS. Therefore the calculation method is at least as useful the application of lactate-concepts to detect MLSS.

In contrast to lactate-concepts, however, by using the calculation method it is also possible to show the influence of individual ${\dot{V}O}_{2\max}$ and ${\dot{V}\mathrm{La}}_{\text{max}}$ on MLSS, as well as their combined effects. This can be highlighted for subjects with similar ${\dot{V}O}_{2\max}$ values, for example subject 5 and 12 at 61.0 and 62.7 ml·min^−1^·kg^−1^ respectively. Using the classical interpretation, endurance performance of these subjects would be nearly the same, yet interestingly PMLSS_E_ of subject 5 and 12 were completely different (172 vs. 278 W). To explain this difference of 106 W, it is not possible to use only ${\dot{V}O}_{2\max}$, but differences in ${\dot{V}\mathrm{La}}_{\text{max}}$ of both subjects (1.39 vs. 0.94 mmol·l^−1^·s^−1^) is also required. Therefore, subject 5 produces significantly more lactate within the muscle cell per second in contrast to subject 12. When related to the same ${\dot{V}O}_{2\max}$, this higher lactate production rate leads to a reduction of MLSS [10,12].

On the other hand it also seems pertinent to focus on subjects with the same PMLSS_E,_ for example subject 5 and 13 (172 vs. 180 W). It is essential to mention that ${\dot{V}O}_{2\max}$ and ${\dot{V}\mathrm{La}}_{\text{max}}$ values of these subjects are completely different (61 vs. 49 ml·min^−1^·kg^−1^ and 1.39 vs. 0.81 mmol·l^−1^·s^−1^, respectively). This particular example explains, why individuals with the same MLSS could originate by completely different combinations of ${\dot{V}O}_{2\max}$ and ${\dot{V}\mathrm{La}}_{\text{max}}$ as previously suggested by Bleicher et al. [12]. Therefore the knowledge of ${\dot{V}O}_{2\max}$ and ${\dot{V}\mathrm{La}}_{\text{max}}$ and the application of the calculation method could help for a better interpretation of MLSS.

### Limitations

The reason for the overestimation of PMLSS_C_ is likely caused by methodological as well as physiological aspects related to its calculation. It is well known, that a high positive correlation between ${\dot{V}O}_{2\max}$ and PMLSS exists, which incidentally was confirmed in the present study, and highlights the importance of ${\dot{V}O}_{2\max}$ concerning PMLSS. The determination of ${\dot{V}O}_{2\max}$ is a valid test procedure and well established in performance and clinical diagnostics [23]. However, Mader and Heck [3], Bleicher et al. [12], Heck and Schulz [14] and Mader [11] showed that on a theoretical basis, ${\dot{V}\mathrm{La}}_{\text{max}}$ must have a significant influence on PMLSS. In the present investigation ${\dot{V}\mathrm{La}}_{\text{max}}$ shows no correlation with PMLSS_E_, which was probably caused by the small range of ${\dot{V}\mathrm{La}}_{\text{max}}$ values measured in this investigation. Furthermore, the missing correlation between ${\dot{V}\mathrm{La}}_{\text{max}}$ and PMLSS_E_ as well as the overestimation of PMLSS_C_ may have been caused by the methodological procedure in determining the maximal anaerobic performance. For example, in the present study ${\dot{V}\mathrm{La}}_{\text{max}}$ was measured by a sprint-test lasting 15 s. It is possible that testing ${\dot{V}\mathrm{La}}_{\text{max}}$ by using a test duration lower than 15 s would lead to higher maximal glycolytic rates and therefore on the basis of the same ${\dot{V}O}_{2\max}$ to a lower PMLSS [3,12,14]. Hauser [24] showed, that ${\dot{V}\mathrm{La}}_{\text{max}}$ increases by 8% when measured using a 13 s sprint-test compared to a 15 s sprint-test. If the present ${\dot{V}\mathrm{La}}_{\text{max}}$ of 0.91 mmol·l^−1^·s^−1^ would be increased by 8%, the PMLSS_C_ would have been 224 W. The bias between PMLSS_C_ and PMLSS_E_ would only be −7 W, which could from a practical point of view be neglected. Therefore test procedures of ${\dot{V}\mathrm{La}}_{\text{max}}$ must receive greater focus in future investigations.

Another reason for the differences between the two methods could be the defined interval of 10 W between two constant-load tests, which was used because of time and economic reasons. Using the interval of 10 W it is possible, that PMLSS_E_ is underestimated by a mean by 4 - 5 W. Consequently, it is possible that PMLSS_E_ does not represent the PMLSS exactly. The possible increase of PMLSS_E_ of 4 - 5 W would lead to a decrease in the difference between PMLSS_C_ and PMLSS_E_ of −7 W.

In addition, physiological reasons for differences could be based on the biological variability of the parameters and constants that were used in the calculation. As pointed out by Mader and Heck, the relation between ${\dot{V}O}_{2}$ and power output (Ks4) has an important influence on PMLSS [3]. Again according to Mader and Heck [3] Ks4 was set to 11.7 O_2_/W in the present study. This relation corresponds exactly to the determined mean value of Ks4 used with the cycle ergometer. However, Ks4 varies on an interindividual basis [3], and only a theoretical increase of Ks4 by 2.5% would lead to a 224 W decrease in PMLSS_C_. In addition, the day-to-day variability of ${\dot{V}O}_{2\max}$ and ${\dot{V}\mathrm{La}}_{\text{max}}$ also has important influences on PMLSS, with a mean within-subject variation of 5.6% of ${\dot{V}O}_{2\max}$ leading to deviations in PMLSS_C_ of ± 30 W [25]. In contrast, the biological variability of ${\dot{V}\mathrm{La}}_{\text{max}}$ still remains unknown.

## Conclusion

The mathematical method introduced by Mader and Heck [3] for the determination of PMLSS represents an accurate method similar to that of previous lactate-threshold concepts. In contrast to lactate-threshold concepts, however, this novel calculation method is based on ${\dot{V}O}_{2\max}$ and ${\dot{V}\mathrm{La}}_{\text{max}}$ that can be used for explaining the origin of PMLSS and therefore the metabolic response. The knowledge of both parameters, as well as their individual influence on MLSS, could be important for establishing training recommendations, which could lead to either an improvement in ${\dot{V}O}_{2\max}$ or ${\dot{V}\mathrm{La}}_{\text{max}}$ by performing high intensity or low intensity exercise training, respectively.

### Ethical standards

The experiments comply with the current laws of the country. The study was proved by Ethics Commission.

## Abbreviations

ADP: Adenosine diphosphate; AMP: Adenosine monophosphate; ATP: Adenosine triphosphate; AT: Anaerobic threshold; BLC: Blood-lactate-concentration; BW: Body weight; CLa_rest_: Blood-lacate-concentration during rest; CP: Crossing point; Dmax method: Lactate threshold concept; IAT: Individual anaerobic threshold; Ks1: 50%-activity constant of oxidative phosphorylation; Ks2: 50%-activity constant of glycolysis; Ks4: Oxygen/workload equivalent; MaxPostLa: Maximum post excercise blood lactate concentration; MLSS: Maximal lactate-steady-state; MLSSc: Calculated maximal lactate steady-state; MLSS_E_: Experimental maximal lactat steady-state; OBLA: Onset of blood lactate accumulation; PMLSS: Power in maximal lactate-steady-state; PMLSS_C_: Power in calculated maximal lactate-steady-state; PMLSS_E_: Power in experimental maximal lactate-steady-state; PFK: Phosphofructokinase; P_max_: Maximal power; rpm: Revolutions per minute; RER: Respiratory exchange ratio; SD: Standard deviation; t_alac_: Alactic time intervall; ${\dot{V}\mathrm{La}}_{\text{max}}$: Maximum lactate production rate; ${\dot{V}\mathrm{La}}_{\mathrm{ss}}$: Gross lactate formation/activation of glycolysis; ${\dot{V}\mathrm{La}}_{\mathrm{oxmax}}$: Maximal elimination-rate of lactate; ${\dot{V}O}_{2\mathrm{ss}}$: Activation of oxidative phosphorylation; ${\dot{V}O}_{2\max}$: Maximum oxygen uptake; ${\dot{V}\mathrm{La}}_{\mathrm{ssnet}}$: “Net” lactate formation

## Competing interest

The authors declare that they have no conflict of interest.

## Authors’ contributions

Data collection: TH, JA, Manuscript: TH, JA, HS. All authors read and approved the final manuscript.

## References

[B1] Heck H Beneke R 30 Years of Lactate Thresholds – what remains to be done? Dt Z Sportmed 2008 59 297 302

[B2] Beneke R Methodological aspects of maximal lactate steady state-implications for performance testin Eur J Appl Physiol 2003 89 1 95 99 10.1007/s00421-002-0783-1 12627312

[B3] Mader A Heck H A theory of the metabolic origin of "anaerobic threshold" Int J Sports Med 1986 7 1 45 65 3744647

[B4] Heck H Laktat in der Leistungsdiagnostik 1990 Schorndorf: Hofmann

[B5] Hauser T Adam J Schulz H Comparison of selected lactate threshold parameters with maximal lactate‒steady‒state in cycling Int J Sport Med 2013 Epub ahead of print 10.1055/s-0033-1353176 24227122

[B6] Jones AM Doust JH The validity of the lactate minimum test for determination of the maximal lactate steady state Med Sci Sports Exerc 1998 30 8 1304 1313 10.1097/00005768-199808000-00020 9710874

[B7] Sahlin K Harris RC Nylind B Hultman E Lactate content and pH in muscle obtained after dynamic exercise Pflugers Arch 1976 367 2 143 149 10.1007/BF00585150 13343

[B8] Sjödin B Jacobs I Onset of blood lactate accumulation and marathon running performance Int J Sports Med 1981 2 1 23 26 10.1055/s-2008-1034579 7333732

[B9] Dickhuth H-H Yin L Niess A Röcker K Mayer F Heitkamp HC Horstmann T Ventilatory, lactate-derived and catecholamine thresholds during incremental treadmill running: relationship and reproducibility Int J Sports Med 1999 20 2 122 127 10190774 10.1055/s-2007-971105

[B10] Mader A Eine Theorie zur Berechnung der Dynamik und des steady state von Phosphorylierungszustand und Stoffwechselaktivität der Muskelzelle als Folge des Energiebedarfs 1984 Köln: Dt. Sporthochschule

[B11] Mader A Glycolysis and oxidative phosphorylation as a function of cytosolic phosphorylation state and power output of the muscle cell Eur J Appl Physiol 2003 88 4–5 317 338 12527960 10.1007/s00421-002-0676-3

[B12] Bleicher A Mader A Mester J Zur Interpretation von Laktatleistungskurven - experimentelle Ergebnisse mit computergestützten Nachberechnungen Spectrum der Sportwissenschaften 1998 10 92 104

[B13] Harriss DJ Atkinson G Update–Ethical standards in sport and exercise science research Int J Sports Med 2011 32 11 819 821 22065312 10.1055/s-0031-1287829

[B14] Heck H Schulz H Diagnostics of anaerobic power and capacity Dt Z Sportmed 2002 53 202 212

[B15] Mader A Liesen H Heck H Phillipi H Rost R Schürch P Hollmann W Zur Beurteilung der sportartspezifischen Ausdauerleistungsfähigkeit im Labor Dt Z Sportmed 1976 27 80 88 109–112

[B16] Chance B Williams GR Respiratory enzymes in oxidative phosphorylation. I. Kinetics of oxygen utilization J Biol Chem 1955 217 1 383 393 13271402

[B17] Mader A Heck H Mader A, Allmer H Energiestoffwechselregulation, Erweiterungen des theoretischen Konzepts und seiner Begründungen. Nachweis der praktischen Nützlichkeit der Simulation des Energiestoffwechsels Brennpunktthema Computersimulation: Möglichkeiten zur Theoriebildung und Ergebnisinterpretation 1996 Sankt Augustin: Academia-Verl 124 162

[B18] Newsholme EA Start C Regulation in metabolism 1973 London: Wiley & Sons

[B19] Barstow TJ Buchthal SD Zanconato S Cooper DM Changes in potential controllers of human skeletal muscle respiration during incremental calf exercise J Appl Physiol 1994 77 5 2169 2176 7868430 10.1152/jappl.1994.77.5.2169

[B20] Krause U Wegener G Control of adenine nucleotide metabolism and glycolysis in vertebrate skeletal muscle during exercise Experientia 1996 52 5 396 403 10.1007/BF01919306 8641374

[B21] Donovan CM Brooks GA Endurance training affects lactate clearance, not lactate production Am J Physiol 1983 244 1 83 92 10.1152/ajpendo.1983.244.1.E83 6401405

[B22] Van Schuylenbergh R Vanden Eynde B Hespel P Correlations Between Lactate and Ventilatory Thresholds and the Maximal Lactate Steady State in Elite Cyclists Int J Sports Med 2004 25 06 403 408 10.1055/s-2004-819942 15346226

[B23] Weltman A Snead D Stein P Seip R Schurrer R Rutt R Weltman J Reliability and Validity of a Continuous Incremental Treadmill Protocol for the Determination of Lactate Threshold, Fixed Blood Lactate Concentrations, and V̇O_2max_ Int J Sports Med 1990 11 01 26 32 10.1055/s-2007-1024757 2318561

[B24] Hauser T Untersuchungen zur Validität und Praktikabilität des mathematisch bestimmten maximalen Laktat-steady-states bei radergometrischen Belastungen [abstract] 2012 TU-Chemnitz: Chemnitz

[B25] Katch VL Sady SS Freedson P Biological variability in maximum aerobic power Med Sci Sports Exerc 1982 14 1 21 25 10.1249/00005768-198201000-00004 7070252

