# Erratum to: ‘Comparison of calculated and experimental power in maximal lactate-steady state during cycling’

**DOI:** 10.1186/s12976-016-0044-3

**Published:** 2016-09-08

**Authors:** Thomas Hauser, Jennifer Adam, Henry Schulz

**Affiliations:** 1 Chemnitz University of Technology, Chemnitz, Germany; 2 Department of Internal Medicine/Cardiology, University of Leipzig, Heart Centre, Leipzig, Germany

## Erratum

Unfortunately, the original version of this article [1] contained errors. In equation 7, where ADP-concentration is calculated based on VO2ss and VO2max (Calculation of free ADP-concentration with respect to activated oxidative phosphorylation and maximal oxygen uptake): the term “Ks2” is being changed to “Ks1” (Ks2 is wrong within the equation).

In the sentence before equation 7:

**Wrong (current) version:**

"If V˙VOss is known or easily fit from 1 to VO2max V˙O2max, Equation 2 can be rearranged in Equation 7. Therefore ADP-concentration can be calculated for a special workload depending on VO2ss V˙O2ss and VO2max V˙O2max, in the form of…"

**Correct version:**

The number is changing from equation 2 to equation 3 as shown below:

"If VOss V˙O2ss is known or easily fit from 1 to VO2max V˙O2max, Equation 3 can be rearranged in Equation 7. Therefore ADP-concentration can be calculated for a special workload depending on VOss V˙O2ss and VO2max V˙O2max, in the form of…"
